# The Poor Man's Home.—IV

**Published:** 1897-09-11

**Authors:** 


### Modern Sociology.

### THE POOR MAN'S HOME.—IV.

Municipalities can, generally, borrow money more advantageously than private individuals or companies. Indeed, when a large area has to be cleared and rebuilt at a cost of hundreds of thousands, it would be often impossible for private associations to obtain the requisite capital, whereas corporations with the security of the rates at their back can easily obtain it, and that on the most advantageous terms. When, therefore, large areas have to be dealt with, the enterprise is suitable only for public bodies. As also their sole motive is the public welfare—though one would never have them ignore commercial sanity in their movements—they are more strictly bound than individuals or joint stock companies can be, to see that they do not demolish so rapidly as to aggravate much the evil of over-crowding in the neighbourhood—they cannot avoid doing so to a certain extent—and also to see that they provide, although in more wholesome fashion, for the same class as they dispossess. It is only too common to pull down the home of the man who earns £1 a week in order to erect one for the man who earns £2. But it is the very poor who most need that public bodies should provide them with houses, decent and sanitary, at reasonable rents. A rent must be paid that clears the interest on borrowed money, and gives some small percentage beyond, or your casual worker becomes unconsciously a casual pauper; and his employer, who can get his labour cheaper because he can get so cheap a house, is a casual pauper also, profiting, however indirectly, by the taxes drawn from the whole community. This is not the place to enter upon the immensely difficult question of the relation of rich and poor; but we must reiterate the principle that while a municipality, a parish, a county council may rightly accept a pecuniary loss in view of benefit to be obtained by the whole town, or parish, or county, they should not do anything which will, directly or indirectly, benefit individual employers. Thus, they may put up five houses on ground which has hitherto held ten, thus losing half the price of the site; but these five houses being erected, they should not let them at rents which will not be moderately remunerative. The compensation for what they lose on the site must be found in insisting on cleanliness and decency on the part of the tenants, and forbidding over-crowding, thus teaching by degrees a higher standard of living. They should, in the interests of public health and morality, try thus to compete with the rack-renter, the house-sweater, and may directly forbid that taking of lodgers in unrestricted numbers which is so ruinous to health and decency among the poor; but it is not their business to interfere with men who, having ventured their capital, ask only a moderate return for their money. What that return should be cannot be stated in terms of precision; so much depends on the relative value of money in different places. Three per cent. may be enough in London, and 6 per cent. not too much in New York. The only rule that one can lay down is that when the houses of the poor are paying a much higher percentage than those of the well-to-do, they are being too highly rented. This is too often the case, and it is to cure such injustice that one wishes to see public bodies become the landlords of the poorer class.

It is always open to employers of labour to provide houses for their workers, and many do so; some of them at rentals which show that they are meant chiefly as an addition to wages, expended in a wiser form than the worker might spend it himself. Of these we shall speak later. Here we wish only to say that what is always reasonable becomes in certain cases a duty.

Certain philanthropists, who recognise the evils of great towns, but whose outlook goes no further, are always crying out to have factories built in country places. Without going into all the objections to this, it is proper to say that to do so lays upon the master a moral duty to provide houses for those whom he employs. Now to do so implies the expenditure of a very considerable amount of money beyond that required for starting the work itself, and an employer may be excused for hesitating to add this to the cost of a venture of which the success is, at the initial stage, problematical. In some industries, such as mining, which must often be carried on in remote districts, the master must provide houses, and in these circumstances he often provides very bad ones. The excuse is that while he does not know how the venture will result, the owner of the mine or the company that owns it—great is that impersonality, the limited liability company!—must hesitate to spend much on houses which may be occupied only for a short time. So valid is this plea that one may accept it as reasonable; yet it is apt to breed a habit of careless housing in the employer, and an acceptance of bad accommodation in the employed. And when fortune is favourable and the work becomes permanent, these ill-built houses become a very profitable speculation. Allowing for all faults of habit and character in the occupants, it must be owned that the dreary look of many mining villages—the slum stranded on a country road which goes by that name—is due to the meanness with which the houses are built. No conveniences to make decency possible, no garden to teach a love of beauty, to give healthy employment for leisure hours, or even to add variety inexpensively to the daily bill of fare; only a chilly room, hardly raised above the ground, a cramped bed-space, a smoky grate; these are poor materials for a home. Wherever miners are compelled for any reason to become their own landlords you find a better class of house, a higher ideal of comfort, and—whether it be cause or effect—a higher type of man.

Co-operative societies have not yet done much directly in providing houses for their members, but in some of the larger ones they might very conveniently undertake such work, and might thereby benefit their members very considerably. Indirectly, however, their action may be regarded as helpful. Not long ago, at a business meeting of pawnbrokers, the chairman remarked that formerly they had a busy time about quarter-days with people pledging goods in order to raise money for rent. This periodic activity had now ceased, and the speaker traced the change to the action of co-operative societies. Many a man who had not moral strength enough to put away, week by week, the proper proportion of the quarter's rent, could, by buying all his necessaries at the co-operative store, receive enough in dividends when rent-time came round to pay that also without inconvenience.

